# What are ambulance crews’ experiences of using a mechanical chest compression device for out-of-hospital resuscitation? A constructivist qualitative study utilising online focus groups

**DOI:** 10.29045/14784726.2022.09.7.2.24

**Published:** 2022-09-01

**Authors:** Laura Blair, Richelle Duffy

**Affiliations:** North East Ambulance Service NHS Foundation Trust ORCID iD: https://orcid.org/0000-0001-9846-9429; Northumbria University ORCID iD: https://orcid.org/0000-0002-7180-8707

**Keywords:** mechanical chest compressions, pre-hospital, qualitative research

## Abstract

**Introduction:**

Mechanical chest compression devices (MCCDs) provide chest compressions mechanically to a person in cardiac arrest. Those chest compressions would usually be provided manually. Previous studies into the use of MCCDs have focused on the quantitative outcomes, with little emphasis on the qualitative experiences of those using MCCDs.

**Purpose:**

To collect and report ambulance crews’ experiences of using MCCDs for out-of-hospital resuscitation attempts.

**Methods:**

The philosophical approach was constructivist, the methodology qualitative and the data collection method online focus groups. Convenience sampling was used to recruit participants who met the inclusion criteria, which broadly were to have experience of using MCCDs for out-of-hospital resuscitation. There have been two types of MCCD used locally. Participants were included regardless of which type of device they had experience of. Similarly, participants were included whether they had active or passive experience of the devices. The focus groups were recorded, fully transcribed and then analysed using constant comparison.

**Results:**

Four selective codes emerged. These were factors directly affecting ambulance crew members; practicalities of a resuscitation attempt; ambulance crew members’ perceptions, experiences and thoughts; negatives of MCCDs.

**Conclusion:**

The main perceptions arising from the participants’ discussion in this work were that MCCD use could potentially provide psychological protection to ambulance crew members when reflecting on resuscitation attempts, and participants felt there is an overall reduction of cognitive load for ambulance crew members when using MCCDs for resuscitation attempts. There were particularly timely benefits expressed of MCCDs easing the physical fatigue of a resuscitation attempt when responding wearing personal protective equipment, as has been required during the COVID-19 pandemic. MCCDs were felt to be of benefit when transporting a patient in cardiac arrest but differences were expressed as to whether the LUCAS-2 in particular helps or hinders extrication of a patient.

## Introduction

Chest compressions during a resuscitation attempt have traditionally been provided by hand (manually), and the challenges of maintaining high-quality chest compressions in the dynamic pre-hospital environment are well documented (Gates et al., 2015). More recently, mechanical chest compression devices (MCCDs) have been introduced (Wik, 2000), with two main types used; a piston-type device and load distributing band. Findings from several large randomised quantitative studies, synthesised into meta analyses by Gates et al. (2015), updated by Zhu et al. (2019), demonstrated that rates of survival, albeit different measures of survival in different studies, were no different when patients were treated with manual or mechanical compressions.

The Resuscitation Council provides guidelines for resuscitation in the United Kingdom (Resuscitation Council UK, 2021) and currently advises that MCCDs should only be used if high-quality manual chest compressions are not practical or compromise provider safety (Soar et al., 2021). This is further supported by the most recent Cochrane review on MCCDs, which acknowledges that they could be an alternative when high-quality manual compressions are not possible or are dangerous (Wang & Brooks, 2018). Previous work has investigated the specific uses of MCCDs in the pre-hospital environment, such as providing high-quality cardiopulmonary resuscitation (CPR) while extricating (Chen et al., 2021; Lyon et al., 2015) and transporting a patient from scene to the hospital (Fox et al., 2013). Based on these benefits, such devices are still used by some systems, including North East Ambulance Service, which encompasses both urban and rural regions and serves a population of almost three million people (North East Ambulance Service NHS Foundation Trust, 2018).

From a user perspective, parallel research comparing the cognitive and physical demands on helicopter crews when using an MCCD versus providing manual chest compressions found reduced heart rates and better cognition test scores when an MCCD was used (Rehatschek et al., 2016). Anecdotes from an American qualitative retrospective study (Satterlee et al., 2013) suggested that users of MCCDs found that they could be more attentive to other aspects of care. A part of Pocock et al. (2016) asked, via online survey, ambulance staff perspectives on contributing to a national trial of a piston-type device (Perkins et al., 2015). Advantages cited of the LUCAS 2™ mechanical chest compressions (Jolife AB/Stryker, Lund, Sweden, hereafter referred to as LUCAS-2) were provision of quality and consistent compressions and being able to continue chest compressions while extricating downstairs and transporting patients. However, 13% of respondents had never used or seen the LUCAS-2 in clinical practice, meaning some of the data could have been skewed by this lack of experience.

As described, there is emerging evidence that MCCDs appear to address some of the practical and personal challenges faced by clinicians when providing continuous high-quality chest compressions in the pre-hospital environment. Yet, the views and experiences of those ambulance crew members using MCCDs have seldom been robustly sought.

Therefore, the primary aim of this current research was to collect and report ambulance crews’ experiences of using MCCDs for out-of-hospital resuscitation attempts. The secondary aims were to explore whether the use of MCCDs has any effect on, or facilitates, the team leader role at an out-of-hospital resuscitation attempt, and to explore whether providing mechanical versus manual chest compressions has an effect on how ambulance crews feel physically and emotionally after an out-of-hospital resuscitation attempt.

## Methods

The Standards for Reporting Qualitative Research guidelines were followed when writing this article. This study has been registered with ClinicalTrials.gov, ID NCT04478786.

The research methodology was shaped by the principles of constructivist grounded theory (Charmaz, 2013; Creswell & Creswell, 2018). Grounded theory methods were used during the data collection phases to support the collection of rich detailed data, viewed through the lens of the participants’ situational, social and professional contexts. Also, within the data analysis phases, initial line-by-line coding and focused coding were used to categorise the data and identify the adequacy of the codes. The process of prolonged active engagement was used to further assess the quality, credibility and sufficiency of the findings grounded in the reality of the subjects, rather than to generate a substantive theory.

The researcher is an emic researcher, credible to conduct this work (Polit & Beck, 2012), who designed a high-quality, robust and relevant study, using their existing relationship with participants to facilitate inclusive engagement in the focus groups, while being critically self-reflective of the influence their own presence and behaviours could have on the data gathered (Etherington, 2004). Support for the design of this work was provided by the academic supervisor and experienced academic ambulance service colleagues.

Convenience sampling was used to recruit operational employees of the local ambulance trust who met the inclusion criteria of experiencing MCCDs at an out-of-hospital resuscitation attempt. Participants were recruited whether they had experience of either of the two types of MCCD used in this trust and whether they had active or passive experience of MCCDs. Active involvement was classed as being responsible for the decision to use or physically deploying the device, including during patient recruitment to Perkins et al. (2015), or responding as a current critical care response or specialist paramedic – emergency care. Passive involvement was classed as any ambulance crew member who was involved in an out-of-hospital resuscitation attempt where another crew or resource provided an MCCD, and they assisted with or observed its use but weren’t responsible for it.

It was accepted that some participants would not have had active involvement with MCCDs for a few years, as recruitment to Perkins et al. (2015) ended in 2013. However, it was deemed important to still include those participants, as well as both generalist and specialist roles, to gain a breadth of experiences. Morgan (1998) advises that groups with homogeneity in shared experience require less time to build a rapport. It was anticipated that the shared experience of attending resuscitation attempts would encourage spontaneous conversation (Acocella, 2012).

University of Northumbria ethics panel and Health Research Authority (HRA) approval were gained. The participant information sheet, consent and demographics forms were emailed to the participants after they expressed an interest in the study. Consent was gained from all participants. A gift voucher was offered to participants, deemed to acknowledge their efforts and show reciprocity, but not intended to exploit them (Creswell & Creswell, 2018).

The method of data collection was focus groups, with the evidence suggesting these are ideal for exploring people’s experiences and ways of thinking (Kitzinger, 1995). The occurrence of the COVID-19 pandemic during the planning of this study necessitated a change from initially planned face-to-face focus groups to synchronous audio-visual online ones. This was deemed reasonable and pragmatic. Microsoft Teams was used to host these groups and the audio was recorded onto a separate dictaphone.

The emic researcher facilitated the focus groups, with a second facilitator for support. A topic guide was developed from the literature and researchers’ understanding of the subject. It was not formally piloted but face-validity was checked by other members of the research team. The topic guide provided a basis for conversation but each focus group took on a bespoke direction of questioning based on the emergent discussion. Not every question was used in each focus group and there were some questions that occurred organically and so do not appear in the topic guide. In line with an emergent style, the topic guide was then further developed for each subsequent focus group, based on discussion generated in the previous one (Morgan, 1997). The topic guide is included as Supplementary 1. The standby ranking activity on the topic guide was not required as the conversation never stalled.

The transcription of the focus groups was subcontracted to a secure third-party secretarial service. The researcher repeatedly listened to and got immersed in the audio recordings, engaging in the process of constant comparison across the transcripts, as described by Onwuegbuzie et al. (2009). This led to open, axial and selective codes being generated. Findings between each of the focus groups were compared and contrasted for congruence.

Trustworthiness in the data was ensured by allowing the participants’ answers to naturally emerge, including anonymous quotes from the participants, providing a clear audit trail of findings and having the academic supervisor code a portion of the data to ensure inter-coder reliability. Member checking of relaying the emergent themes from each focus group to the involved participants also occurred.

## Results and discussion

The focus groups sample size ranged from two (due to a late cancellation) to five, and the four groups were held between July and September 2020. The total sample recruited was 15. Participants’ clinical experience encompassed the roles of advanced paramedic, specialist paramedic – emergency care, paramedic, newly qualified paramedic, student paramedic, emergency care technician, clinical care assistant and clinical care manager. Participants’ experience was largely with the LUCAS-2 (a piston device). Seven participants had experienced the AutoPulse (a load-distributing band) but only one of those seven had active experience within an ambulance service environment. Of the 15 participants, 11 had passive experience only with either device. Demographic information is missing for one participant. Summary demographics are presented in Table 1.

**Table 1.** Summary of participant demographics.

	Median	Range	IQR
Duration of operational experience (years)	7	< 1 year–28	6
Estimated number of episodes of MCCD experience	6	1–100	8
Approximate time since last experience (days)	28	Last few days–672	35

IQR: interquartile range; MCCD: mechanical chest compression device.

A total of 140 open codes were identified, provided as Supplementary 2. Onwuegbuzie et al. (2009) describe open coding as chunking the data into small units, the second stage of axial coding being to group these open codes into categories, culminating in the final phase of selective coding which is to create overarching themes. Novel axial codes continued to emerge when coding the fourth focus group, suggesting that saturation of data had not yet been reached. Approvals given for this work were limited to four focus groups. Final coding identified 18 axial codes and four selective codes, presented here as Table 2.

**Table 2.** Selective and axial codes.

Selective codes	Axial codes
Factors directly affecting ambulance crew members	Physical benefits to ambulance crew members
	Benefits to doing resuscitation in PPE
	Facilitates cognition for ambulance crew members
	Psychological protection for ambulance crew members
	Effect on empathy
Practicalities of a resuscitation attempt	Equivalent to an extra ambulance crew member
	Effect on team leader role
	Facilitates transport of patients in cardiac arrest
	Factors regarding extrication
Ambulance crew members’ perceptions, experiences and thoughts	Personal experiences
	Belief of high-quality compressions / better patient outcomes
	Future deployment suggestions
Negatives of MCCDs	Device appearance, design and size limitations
	Device comparison
	Design suggestions
	Increased hands-off fraction (duration of time with no chest compressions) if prolonged deployment / untrained crew
	Caution with reliance on MCCD
	Ambulance crew unfamiliarity with MCCD, increasing cognitive demands on specialist paramedic

MCCD: mechanical chest compression device; PPE: personal protective equipment.

The four selective codes will be explored in detail. MCCD is a generic term and where there is specific mention made of a particular device this will be made explicit.

### Factors directly affecting ambulance crew members

Most participants referred to MCCDs reducing physical exertion and fatigue, with specific benefits identified of using them when wearing level-3 personal protective equipment (PPE) for resuscitation, as is required during the COVID-19 pandemic. One participant compared resuscitations both with and without an MCCD during the current pandemic by saying, ‘Not having the automatic – the LUCAS … particularly over a prolonged arrest, it makes a massive difference … how well you can continue to do CPR’ (focus group (FG)1).

MCCDs were described as allowing thinking space and increasing cognitive bandwidth, and resuscitations were described as less stressful, with the same phrase of ‘one less thing to think about’ (FG1 and FG4) being used by both paramedics and non-paramedics. A novel notion offered by one paramedic was that the use of LUCAS-2 reduces the amount of audible CPR feedback, which other participants of that focus group agreed to be distracting to their cognition. This audible feedback is of the quality of the chest compressions being provided, detected by a sensor placed on the patient’s chest and provided audibly, and visually, by the defibrillator machine. Most participants described MCCDs as removing the need to actively monitor and manage manual CPR quality, with one participant describing ‘cognitive … able to push the CPR just on to that device … not having to think about it, it’ll go through its cycle … you can focus on those other things’ (FG2). The participants’ perceptions expressed here of the potential cognitive protection provided by MCCDs echo those found by Rehatschek et al. (2016).

At a resuscitation where LUCAS-2 was felt to be a positive addition, a paramedic with passive experience of both device types recalled ‘I could walk away feeling in my mind that they’d had every best possible chance’ (FG3). This anecdote suggests that MCCD use could offer an emotionally protective factor for crews’ processing of and reflection on resuscitation attempts. This paramedic felt that cumulatively over their career this would lead to ultimately less psychological burden. A clinical care assistant compared reflecting on resuscitation attempts by saying: ‘the end of an arrest … you’re thinking … “I hope I did good enough CPR” … whereas if you had that device it’s set … it’ll do it all automatically and you literally don’t have to worry or think about that’ (FG2).

This potential psychological and emotional protection is an important feature of these findings, given the recognition of the psychological effects of traumatic events on ambulance crew members (Bennett et al., 2005), particularly those individuals’ perception and interpretation of such events (Mildenhall, 2019).

A novel notion expressed by a clinical care assistant in one focus group was that using equipment removes empathy but also that the use of machines allows some distance from the outcome of the resuscitation. When asked whether this was a positive or negative factor, the participant expressed conflicting emotions: ‘I don’t know, both really isn’t it … do you want to take all your jobs home or do you feel like you need a bit more empathy in your work’ (FG4).

A paramedic with passive experience of both device types offered a different viewpoint: ‘you don’t feel a sense of accomplishment because it was the machines … that got the ROSC (return of spontaneous circulation)’ (FG4). To complement the previous section, these participants’ comments seem to suggest that MCCDs can offer some degree of protection to an ambulance crews’ emotional processing after an unsuccessful resuscitation, while at the same time diminishing a sense of achievement if there is a positive outcome.

### Practicalities of a resuscitation attempt

Phrases suggesting that MCCDs free up a set of hands were used in all four of the focus groups, along with the positive notion that MCCDs replace team members. One participant explained: ‘CPR device … freeing up two extra people … otherwise would’ve been taking turns to do CPR’ (FG3). When comparing team-leading a resuscitation with and without a MCCD, a paramedic described ‘without mechanical CPR it does require more thinking, more processing … by whoever’s leading that arrest’ (FG3).

There was consensus across all focus groups of the specific benefit of MCCDs when transporting a patient, as they allow continuation of chest compressions while on the move: ‘mechanical CPR, excellent if we have to move while delivering it’ (FG3). The current national stance was described earlier, with transport situations being one of the instances in which MCCDs are reasonable to use, in line with findings here.

Differences were expressed around whether MCCDs aid extrication of a patient. A few participants expressed a view that a LUCAS-2 device in particular can hinder extrication, as it is heavy, in addition to the patient, therefore in contrast to the earlier axial code that MCCDs largely help with reducing physical exertion. Two participants did however also recognise that LUCAS-2 provides continuous compressions during extrication, even though the extrication may be a little more difficult. The positive comments of MCCDs facilitating continuous chest compressions during extrication and transport are in line with findings by Lyon et al. (2015), identified in the introduction.

### Ambulance crew members’ perceptions, experiences and thoughts

Almost half of participants felt that MCCDs allowed other interventions to be conducted in a more efficient manner, and one participant with passive experience of both device types said that an MCCD ‘gives us an opportunity and a time to tidy up’ (FG4). There was such strong belief in LUCAS-2 by two participants in particular that when reflecting on an unsuccessful resuscitation it was felt the outcome may have been more positive if a LUCAS-2 device had been used from the beginning: ‘I can’t say whether … might’ve been a different outcome but … it might’ve been a possibility’ (FG2). This relates to the earlier discussion of whether MCCDs are protective of the psychological processing of resuscitations by ambulance crew members.

One specialist paramedic – emergency care, however, felt that MCCD use is situation specific and not always required, expressing that ‘if you’ve got enough people … swapping them over every two minutes … you keep an eye on people’s quality … keep an eye on themselves … it’s just as good to do manual’ (FG1).

There was strong and sustained opinion of MCCDs providing high-quality chest compressions. One paramedic with passive use of LUCAS-2 said using the device was ‘effectively increasing their [the patient’s] chances’ (FG2) because of the effective CPR. Although this belief of MCCDs providing high-quality compressions and potentially better outcomes was a frequently made comment, participants often struggled to articulate the reasons for this. Some of the reasons given were ‘standardised and set, there’s no distraction’ (FG4), ‘frees up other things … correctly, accurately … reliably’ (FG4), ‘absolutely consistent … it will never tire’ (FG3). The views expressed here align with those described earlier by Pocock et al. (2016) of the LUCAS-2.

Both generalist and specialist participants described tailored future use of MCCDs, rather than routine. A paramedic with passive experience of both types of device offered a suggestion of when MCCDs would be appropriate to use: ‘prolonged cardiac arrests … cardiac arrests that you’re going to transfer … cardiac arrests that don’t have a lot of staff there’ (FG3), essentially any resuscitation attempt where there are barriers to the provision of high-quality, uninterrupted chest compressions. It was felt that if there is already adequate spatial access to the patient and adequate crew members, there may not be a role for an MCCD. An MCCD was suggested as being appropriate for rural locations because of the lengthy back-up response times. With regard to the number of ambulance resources at a resuscitation attempt, almost half of the participants viewed that even with an MCCD an extra resource was still required to aid with tasks such as history taking, extrication and device deployment. A few participants did, however, suggest that the use of an MCCD could reduce the number of personnel dispatched to an out-of-hospital cardiac arrest.

### Negatives of mechanical chest compression devices

The negatives expressed included the appearance of them, with one participant saying ‘both of them in two different ways look so dramatic and so brutal’ (FG4). A few participants took the time to explain the purpose of MCCDs to family members or bystanders, seemingly because of an awareness that MCCDs look different to the ‘expected’ manual chest compressions and crews felt an obligation to explain.

The LUCAS-2 was described as being difficult to deploy on larger patients, and although it is heavier to carry it was deemed easier to deploy than the AutoPulse. The AutoPulse exceeds the LUCAS-2 in extrication though. In comparing the two device types, one participant who had active experience of both types of device made comparisons: ‘much stronger believer in the AutoPulse for extrication … the carry sheet … much easier device … don’t have that top-heavy load on top of the patient. It’s a band rather than what the LUCAS is’ (FG1).

There were a small number of design developments suggested, including whether the LUCAS-2 could be adapted to fit into a scoop for stability when extricating or have means of extricating the patient incorporated, like the AutoPulse. It was also felt that the LUCAS-2 needs to be adjustable to fit more patients.

In contrast to the earlier comments of MCCDs being protective in terms of cognition, a limitation for specialist paramedics currently responsible for deploying the devices in this trust is that they often find generalist ambulance crews have limited exposure to and training on deployment of the devices. This can increase the cognitive and leadership demands on that specialist paramedic responsible for the MCCD, with one specialist describing ‘we’re the only ones that are trained on them … trying to team lead … having to be distracted by teaching how to put the LUCAS on’ (FG1). A solution offered by both specialist and generalist participants was for there to be training provided for all operational members of ambulance crew.

## Conclusion

The authors acknowledge that this was a small, exploratory qualitative study which required a late but pragmatic change from the planned face-to-face focus groups to online focus groups because of the occurrence of the COVID-19 pandemic and requirement to socially distance. The study did not set out to quantitatively measure any of the effects of MCCDs as described by participants and there may have been an element of recall bias for some. However, the participants’ main perceptions of MCCDs were the potential psychological protection afforded to ambulance crew members when reflecting on resuscitation attempts where MCCDs have been used, an overall easing of cognitive load on those performing resuscitation with MCCDs and a reduction in physical fatigue when performing a resuscitation with an MCCD when wearing level-3 PPE. There was further support for MCCDs being of specific benefit when transporting a patient with ongoing resuscitation, although differences in opinion were expressed as to whether the LUCAS-2 in particular helps or hinders extrication of a patient. To expand on these findings and develop this work, the views expressed by participants suggest that MCCDs appear to protect against any potential worry about chest compression quality. This appears to be because MCCDs are set to provide consistent chest compressions in line with guidelines. More research is therefore needed to explore this possible psychological benefit for ambulance crew members when an MCCD has been used, in comparison to manual chest compressions, along with whether this has an effect on the later development, or absence, of psychological trauma. This is an important area of research for ambulance crew members in all roles, promoting longevity of careers, reducing the risk of burnout and exploring the increasingly recognised links between post-traumatic stress disorder and the paramedic profession (Baqai, 2020).

From the views expressed by participants, it seems the use of MCCDs can relieve some of the cognitive load of co-ordinating tasks and monitoring manual CPR quality, also making the team leader role easier to perform. The exception is when untrained crews are dispatched alongside a specialist paramedic, meaning the cognitive load can be increased for the specialist. The solution of familiarisation training and increased exposure for all generalist ambulance crews was offered by participants. This was felt to increase crews’ confidence and ability to assist with deploying MCCDs, countering the potential harm caused by delayed deployment and reducing the cognitive burden on those responsible for MCCDs.

Further limitations of this work are that they only include the views of ambulance crew members in the North East region so findings may not reflect a national picture. The use of online focus groups may have meant that those unfamiliar or uncomfortable with technology may have chosen not to participate, although the sample size was still achieved. Likewise, the response to COVID-19 meant this study was advertised via online means only (emails and trust social media), potentially impacting on the sample who volunteered, depending on who saw the advert. Given that this use of synchronous audio-visual online focus groups is a relatively new research method, the researcher could have incorporated seeking feedback from the participants about their experience of these groups, as suggested by Daniels et al. (2019).

In conclusion, these qualitative findings enhance understanding of the use of MCCDs in relation to the cognitive, physical and psychological implications for ambulance crew members using them.

## Acknowledgements

Dr. Graham McClelland provided a wealth of knowledge and assisted with the design and facilitation of the focus groups. Dr. Lee Thompson provided expert opinion on conducting focus groups and development of the topic guide. North East Ambulance Service NHS Foundation Trust acknowledges the support of the National Institute for Health Research Clinical Research Network (NIHR CRN).

## Author contributions

LB was the primary researcher and author, supervised by RD. Both authors contributed to the final manuscript. LB can answer any questions about this work. RD acts as the guarantor for this article.

## Conflict of interest

None declared.

## Ethics

HRA approval was given to conduct this study (IRAS ID: 275607), as well as ethical approval by Northumbria University Ethics panel (submission reference: 18018).

## Funding

Funding for this project was provided by Stryker (Jolife AB/Stryker), Lund, Sweden, Scheelevagen 17, 223 70. This company manufactures one of the types of MCCD discussed in this study. Stryker was not involved in the design or analysis of the study and had no influence on the writing of this article. This article was shared with the funder prior to publication, as stipulated in their funding contract.
